# Gender Differences in Dysfunctional Attitudes in Major Depressive Disorder

**DOI:** 10.3389/fpsyt.2020.00086

**Published:** 2020-02-27

**Authors:** Xuemei Qin, Jinrong Sun, Mi Wang, Xiaowen Lu, Qiangli Dong, Liang Zhang, Jin Liu, Yumeng Ju, Ping Wan, Hua Guo, Futao Zhao, Yan Zhang, Bangshan Liu, Lingjiang Li

**Affiliations:** ^1^Department of Psychiatry, The Second Xiangya Hospital, Central South University, Changsha, China; ^2^Mental Health Institute of Central South University, China National Clinical Research Center on Mental Disorders (Xiangya), China National Technology Institute on Mental Disorders, Hunan Technology Institute of Psychiatry, Hunan Key Laboratory of Psychiatry and Mental Health, Changsha, China; ^3^Affiliated WuTaiShan Hospital of Medical College of Yangzhou University, Yangzhou Mental Health Centre, Yangzhou, China; ^4^Department of Psychiatry, Zhumadian Psychiatric Hospital, Zhumadian, China

**Keywords:** major depressive disorder, dysfunctional attitudes, gender differences, seeking applause, dependence, self-determination attitude, gender-specific interventions

## Abstract

**Background:**

Dysfunctional attitudes play a key role in the development and prognosis of depression. Gender also plays an important role in many clinical features of major depressive disorder (MDD). This study is aimed at investigating the gender differences in dysfunctional attitudes in patients with MDD.

**Methods:**

One hundred and seventy-two patients with MDD and 159 healthy controls (HCs) were enrolled in this study. Dysfunctional attitudes were assessed by the Chinese version of the dysfunctional attitude scale—form A (C-DAS-A) and depression severity was assessed by the 24-item Hamilton rating scale for depression (HAMD_24_). The 14-item Hamilton Anxiety Rating Scale (HAMA_14_) was used to measure anxiety. Factorial analysis of variance (ANOVA) of gender and diagnosis on C-DAS-A total and factor scores was adopted with age, education, and body mass index (BMI) controlled. Multiple linear regression analyses of DAS were performed in the MDD group.

**Results:**

First, the C-DAS-A score in the MDD group was increased significantly than HCs. Second, female patients with MDD showed significantly higher scores in C-DAS-A total and three-factor scores (seeking applause, dependence, and self-determination attitude), while no significant difference between female HCs and male HCs was detected. Third, five variables (age, gender, smoking history, HAMD_24_, and HAMA_14_) had predictive effects on and gender showed the greatest contributions to C-DAS-A total and three-factor scores (seeking applause, dependence, and self-determination attitude).

**Conclusion:**

Females with MDD may be linked to more severe cognitive distortion than their male counterparts in seeking applause, dependence, and self-determination attitude, supporting the reasonableness for gender-specific psychosocial interventions.

## Introduction

According to Beck’s cognitive model, depression results from an interaction of negative thinking styles and stressful events (1). These negative thinking styles are typically conceptualized as dysfunctional attitudes, which are rigid and maladaptive beliefs about oneself, the world, and the future. Previous studies have shown that dysfunctional attitudes play a central role in the development and prognosis of depression (2). Specifically, subjects with dysfunctional attitudes are associated with higher risk and poorer prognosis of major depressive disorder (MDD) (3), including more severe depression (4), poorer response to antidepressant treatment (5), shorter time to (6–8), and higher risk of (6) relapse and/or recurrence.

On the other hand, gender also plays a major role in many clinical features of MDD. The lifetime prevalence of depression in females is twice as that in males (9, 10). Females with MDD tend to show younger age of onset (11), longer duration (12), more severe and recurrent episodes (13), higher comorbidity of anxiety, lower comorbidity of substance use, greater functional impairment (11), and lower quality of life (14) than male patients. In the clinical symptomatology, females with MDD report higher rates of increased appetite and weight gain, somatic concern, and hypochondriasis than their male counterparts (12, 15). Taking the roles of gender and dysfunctional attitudes in depression together, it is of particular interest whether there may as well be gender differences in the dysfunctional attitudes of MDD.

Several studies have investigated gender differences in dysfunctional attitudes in the general population and patients with MDD, but the results are mixed. A study recruiting 644 college students showed that there is no gender difference in the dysfunctional attitude scale (DAS) (16). Two studies found no gender difference in dysfunctional attitudes in depressed patients (17, 18), while the other two studies found more dysfunctional attitudes in female patients than in male patients. Specifically, Farmer et al. (19) found that female patients and their siblings show more dysfunctional attitudes on dependence as compared with their male counterparts. Ou et al. (20) found that female patients with hypertension comorbid with depression show significantly higher scores in five factors (attraction and repulsion, compulsion, seeking applause, dependence, self-determination attitude) and lower scores in one factor (cognition philosophy) of the DAS than male patients. These findings indicate that gender differences in dysfunctional attitudes may vary across samples with different characteristics or diagnoses.

Given the key role of dysfunctional attitudes in MDD and the common gender differences in the clinical features of depression, we conducted this study to investigate gender differences in dysfunctional attitudes in a Chinese adult MDD sample. Based on the poorer prognosis of female MDD patients than male MDD patients, we hypothesized that females with MDD would show more dysfunctional attitudes than their male counterparts.

## Materials and Methods

### Subjects

The data reported in this paper comes from a large-sample, longitudinal project investigating the biological and psychological mechanisms of MDD (hypothalamic-pituitary-adrenal axis function and magnetic resonance imaging study of trauma-related depression, registration number: ChiCTR1800014591). We used structural and functional magnetic resonance imaging (MRI) to investigate the neural substrates of MDD and to assess the correlations between clinical and psychological variables (including dysfunctional attitudes) and neural substrates in MDD. We also assessed the hypothalamic-pituitary-adrenal axis function in MDD patients.

One hundred and seventy-two patients were recruited from the Zhumadian Psychiatric Hospital, Henan, China. The participant enrollment procedure took place from January 2013 to December 2018. Patients were diagnosed by two experienced psychiatrists based on the Structured Clinical Interview for DSM-IV (SCID-IV). The severity of depression was moderate-to-severe with a 24-item Hamilton rating scale for depression (HAMD_24_) total score > 19. Patients meeting the following criteria were excluded: 1) met the diagnostic criteria of other psychiatric disorders excepting for generalized anxiety disorder (GAD); 2) had substance abuse history apart from tobacco dependence; 3) had a history of severe physical condition; and 4) had significant suicide ideation or suicide attempts. One hundred and fifty-nine healthy controls (HCs) with a HAMD_24_ total score < 8 were enrolled from the nearby communities of the Zhumadian Psychiatric Hospital. None of them had a current or lifetime diagnosis of any psychiatric disorders or substance abuse except for tobacco dependence. All of the participants aged 18–55 years. A more detailed description of the inclusion and exclusion criteria of participants has been reported in another paper of this project (21).

The present study was approved by the ethics committee of the Second Xiangya Hospital of Central South University and the ethics committee of the Zhumadian Psychiatric Hospital. Written informed consent was obtained from all of the participants after a detailed explanation of the purpose and procedure of the study.

### Measures

#### Dysfunctional Attitudes

Dysfunctional attitudes are usually assessed by DAS (22). Two forms of DAS have been developed: form A (DAS-A) and form B (DAS-B). Between them, DAS-A is used much more frequently than DAS-B since both of them have similar psychometric properties but DAS-A has much fewer items and takes a much shorter time than DAS-B. In this study, we used the Chinese version of the dysfunctional attitude scale—form A (C-DAS-A), which has shown good reliability and validity in a Chinese MDD sample (23, 24). The C-DAS-A is a self-report scale consisting of 40 items. Each item consists of a statement about the subject and a 7-point Likert scale assessing the extent of agreement (7 = fully agree; 1 = fully disagree). Ten items are scored reversely (item 2, 6, 12, 18, 24, 29, 30, 35, 37, 40). The higher the total score, the more dysfunctional attitudes. In the study of the reliability and validity of the C-DAS-A for Chinese MDD patients, an eight-factor structure (24) of C-DAS-A was proposed and adopted widely in later studies (20, 25, 26), which was also used in the present study. The eight factors are vulnerability (vulnerable self-confidence such as hold an attitude that “People will probably think less of me if I make a mistake”), attraction and repulsion (believe that happiness depends on other’s love, e.g., “If others dislike you, you cannot be happy”), perfectionism (pursuit perfection immoderately like “It is difficult to be happy unless one is good looking, intelligent, rich and creative”), compulsion (selective or overly generalization like “To be a good, moral, worthwhile person, I must help everyone who needs it”), seeking applause (has a rigid tendency to pursue the approval of others like “My value as a person depends greatly on what others think of me”), dependence (lack of self-independence like “If you don’t have other people to lean on, you are bound to be sad”), self-determination attitude (casting one’s value to comparison with others like “If I do not do as well as other people, it means I am an inferior human being”), and cognition philosophy (positive attitudes such as “Happiness is more a matter of my attitude toward myself than the way other people feel about me”). Items categorized into each factor were reported in Chen et al. (24)

#### Depression

Depression was assessed by the HAMD_24_, which is the most widely used clinician-report scale of depression.

#### Anxiety

The 14-item Hamilton Anxiety Rating Scale (HAMA_14_) was used to assess anxiety. The total score of HAMA_14_ > 14 means definite anxiety symptoms.

### Data Analysis

Firstly, two-sample independent t-tests and chi-square tests were used to assess the differences in the demographic information between different groups. Analysis of covariance (ANCOVA) was performed to assess the differences in the clinical information between different groups with unmatched demographic variables as covariance respectively. Secondly, factorial analysis of variance (ANOVA) of gender and diagnosis on C-DAS-A total and factor scores was performed with age, education, and body mass index (BMI) controlled. When there was a significant interaction effect between gender and diagnosis, the simple effects of gender on C-DAS-A total and factor scores were analyzed in the MDD group and HC group separately. Lastly, to investigate the effects of age, gender, education, BMI, smoking history, HAMD_24_, HAMA_14_ predicting dysfunctional attitudes in MDD patients, multiple linear regression analyses were used. Statistical significance was set as a two-tailed P ≤ .05.

## Results

### Demographic and Clinical Information of MDD and HC Groups

Demographic and clinical information of MDD patients and HCs are shown in **Table 1**. In the MDD group, gender differences were shown in age, education, and first episode age (*P_1_* = .026, .045, .027, respectively). There were no differences in BMI, HAMD_24_, and HAMA_14_ (all *P_1_* > .05). In subgroup analyses of MDD patients, 114 patients are moderate MDD and 58 patients are severe MDD. We defined that consuming tobacco in a lifetime or currently smoking any amount as having a smoking history and there are 155 patients with no smoking history and 17 with a smoking history. 126 patients are comorbid with GAD and 46 patients are not comorbid with GAD. No significant difference was found in C-DAS-A total score between subgroups of moderate or severe patients, patients with or without GAD, as well as patients with and without smoking history (**Table S1**). The differences in demographic and clinical information of different severity of MDD patients and HC groups are shown in **Table S2**.

**Table 1:** Demographics and clinical information of major depressive disorder (MDD) and healthy control (HC) groups.

Item	MDD			HC				*P_3_*	
	Male (n = 75)	Female (n = 97)	*P_1_*	Male (n = 74)	Female (n = 85)	*P_2_*			
Age (years)	33.25 ± 9.86	36.55 ± 9.32	**.026**	30.04 ± 8.00	38.48 ± 8.20	**< .001**		.591	
Gender (%)	43.6	56.4	–	46.5	53.5	–		.592	
Education (years)	10.87 ± 3.63	9.79 ± 3.30	**.045**	11.96 ± 3.00	10.35 ± 3.90	**.004**		**.032**	
BMI (kg/m^2^)	21.91 ± 2.97	22.08 ± 3.11	.718	24.53 ± 3.14	22.87 ± 2.62	**< .001**		**< .001**	
First episode age (years)	29.86 ± 9.93	32.30 ± 10.12	**.027**	–	–	–		–	
HAMD_24_	30.89 ± 6.76	32.20 ± 7.70	.482	1.11 ± 1.71	2.02 ± 2.73	.091		**< .001**	
HAMA_14_	17.58 ± 5.47	18.85 ± 6.67	.331	0.89 ± 1.58	1.81 ± 2.37	.206		**< .001**	

Data are presented as mean ± SD; Bold values indicate statistical significance; MDD, major depressive disorder; HC, healthy control; BMI, Body mass index; HAMD_24_, 24-item Hamilton Rating Scale for Depression; HAMA_14_, 14-item Hamilton Anxiety Rating Scale; P_1_ , statistical significance of MDD group; P_2_, statistical significance of HC group; P_3_, statistical significance of MDD and HC groups.

In the HC group, males and females showed significant differences in education, age, and BMI (*P_2_* = .004, < .001, < .001, respectively). Distribution of HAMD_24_ and HAMA_14_ was balanced between males and females (all *P_2_* > .05). No statistical significance was noted in age and gender between MDD and HC groups (all *P_3_* > .05). MDD group had lower education years (*P_3_* = .032) and BMI (*P_3_* < .001). Moreover, patients with MDD showed significantly higher HAMD_24_ and HAMA_14_ total score than the HC group (all *P_3_* < .001).

### ANCOVA of C-DAS-A Total and Factor scores of MDD and HC Groups

**Table 2** shows the ANCOVA of C-DAS-A total and factor scores of MDD and HC groups. Patients with MDD showed higher scores in C-DAS-A and all of its eight factors than that of HCs (all *P* < .001). Female MDD patients had higher mean C-DAS-A total and factor scores than their male counterparts judging by scores only.

**Table 2:** Analysis of covariance (ANCOVA) of Chinese version of the dysfunctional attitude scale—form A (C-DAS-A) total and factor scores of major depressive disorder (MDD) and healthy control (HC) groups.

Item	MDD		HC		*P*
	Male (n = 75)	Female (n = 97)	Male (n = 74)	Female (n = 85)	
**Total score** **Factor scores**	150.17 ± 25.98	160.05 ± 28.98	126.92 ± 29.94	123.73 ± 22.30	**<.001**
Vulnerability	17.25 ± 4.42	18.72 ± 4.67	14.68 ± 4.95	14.81 ± 4.13	**<.001**
Attraction and repulsion	18.12 ± 5.04	18.53 ± 6.25	12.77 ± 5.26	12.93 ± 4.76	**<.001**
Perfectionism Compulsion	18.21 ± 5.29 18.28 ± 4.50	19.19 ± 5.94 19.55 ± 4.62	15.15 ± 5.96 16.15 ± 4.30	14.64 ± 4.06 16.34 ± 3.71	**<.001**
Seeking applause	18.45 ± 5.16	20.40 ± 5.47	16.74 ± 8.41	15.59 ± 4.91	**<.001**
Dependence	18.67 ± 4.00	20.36 ± 5.23	15.78 ± 5.09	14.86 ± 4.15	**<.001**
Self-determination attitude	21.07 ± 5.95	23.03 ± 5.28	18.49 ± 5.53	17.24 ± 4.62	**<.001**
Cognition philosophy	20.12 ± 5.08	20.28 ± 5.62	17.16 ± 5.37	17.33 ± 4.73	**<.001**

Data are presented as mean ± SD; Bold values indicate statistical significance; P showed the difference in the C-DAS-A total and factor scores between MDD and HC groups; ANCOVA, analysis of covariance; C-DAS-A, Chinese version of the dysfunctional attitude scale – form A; MDD, major depressive disorder; HC, healthy control.

### Factorial ANOVA of Gender and Diagnosis on C-DAS-A Total and Factor Scores

**Table 3** presents the main and interaction effects of gender and diagnosis as well as the simple effects of gender in MDD and HC groups separately. As shown in **Table 3**, diagnosis showed main effects on C-DAS-A total and all of the 8-factor scores (all *P* < .001). Gender showed no main effect on C-DAS-A total or any of the 8-factor scores. Gender and diagnosis showed significant interaction effects on C-DAS-A total and 3-factor scores (seeking applause, dependence, and self-determination attitude) (*P* = .029, .033, .020, .004, respectively), with females with MDD showing significantly higher scores on C-DAS-A and three of its factors (seeking applause, dependence, and self-determination attitude; *P* = .035, .036, .028, .021, respectively), while no gender difference shown in C-DAS-A total or any of its factor scores in HCs.

**Table 3:** Factorial analysis of variance (ANOVA) of gender and diagnosis on Chinese version of the dysfunctional attitude scale—form A (C-DAS-A) total and factor scores.

Item	Main effects of diagnosis		Main effects of gender		Interaction effects (gender & diagnosis)		Simple effects of gender	
	F	*P*	F	*P*	F	*P*	MDD *P*	HC *P*
**Total score** **Factor scores**	87.59	**<.001**	0.46	.499	4.79	**.029**	**.035**	.332
Vulnerability	39.00	**<.001**	2.05	.153	1.48	.224	–	–
Attraction and repulsion	76.72	**<.001**	0.06	.807	0.14	.707	–	–
Perfectionism Compulsion	41.50 26.06	**<.001** **<.001**	0.43 0.12	.513 .728	0.82 2.17	.366 .141	- -	- -
Seeking applause	19.17	**<.001**	0.50	.479	4.59	**.033**	**.036**	.358
Dependence	60.31	**<.001**	0.41	.524	5.51	**.020**	**.028**	.271
Self-determination attitude	47.75	**<.001**	0.06	.801	8.41	**.004**	**.021**	.084
Cognition philosophy	16.43	**<.001**	0.01	.908	0.03	.857	–	–

Bold values indicate statistical significance; ANOVA, analysis of variance; C-DAS-A, Chinese version of the dysfunctional attitude scale – form A; MDD, major depressive disorder; HC, healthy control.

We also performed subgroup analyses of gender differences on C-DAS-A total and factor scores adding to the supplementary materials (**Table S3** and **Table S4**). The results of factorial ANOVA of gender and diagnosis on C-DAS-A total and factor scores in patients with moderate MDD and HC groups are shown in **Table S3**. Gender and diagnosis showed significant interaction effects on the self-determination attitude (*P* = .002). The simple effects of gender on the self-determination attitude (*P* = .011) showed that females with moderate MDD had higher self-determination attitude scores than that in males. The results of factorial ANOVA of gender and diagnosis on C-DAS-A total and factor scores in patients with severe MDD and HC groups are shown in **Table S4**. As shown in **Table S4**, gender and diagnosis had significant interaction effects on C-DAS-A total and three factor scores (compulsion, seeking applause, and dependence) (all *P* < .05). The simple effects of gender on the compulsion showed that females with severe MDD had higher compulsion scores (*P* = .021).

### Multiple Linear Regression Analyses of DAS in MDD Group

In **Table 4**, we investigated the effects of age, gender, education, BMI, smoking history, HAMD_24_, HAMA_14_ predicting C-DAS-A total and three-factor scores in the MDD group. In the model of C-DAS-A total score, three variables (gender, smoking history, and HAMD_24_) had statistical significance (*P* = .002, .010, .030, respectively) and they explained 9.9% variation of C-DAS-A total score (R^2^ = .099, *P* = .015). Age, gender and smoking history entered into the model of seeking applause (*P* = .039, .002, .029, respectively) with R^2^ =.102. In the model of self-determination attitude, three variables (gender, smoking history, and HAMA_14_) showed significance (all *P* < .05, R^2^ = .112). Gender contributed most in each model (standardized β = .254, .253, .259, respectively). Although the model of dependence had no significance, gender was the only one showing significance among these variables (*P* = .027).

**Table 4:** Multiple linear regression analyses for assessing effects of age, gender, education, BMI, smoking history, 24-item Hamilton rating scale for depression (HAMD_24_), and 14-item Hamilton anxiety rating scale (HAMA_14_) predicting dysfunctional attitudes in major depressive disorder (MDD) patients.

	Variables	Unstandardized β	Standardized β	T	*P*	R^2^	F	*P*
**C-DAS-A total score**	Age	−.082	−.028	−.350	.727	.099	2.584	**.015**
	Gender	14.349	.254	3.116	**.002**			
	Education	−.108	−.013	−.168	.867			
	BMI	−.227	−.025	−.323	.747			
	Smoking history	19.777	.211	2.624	**.010**			
	HAMD_24_	.836	.218	2.186	**.030**			
	HAMA_14_	−.868	−.191	−1.907	.058			
**Factor scores**								
Seeking applause	Age	−.094	−.169	−2.085	**.039**	.102	2.665	**.012**
	Gender	2.745	.253	3.100	**.002**			
	Education	−.037	−.024	−.299	.765			
	BMI	−.007	−.004	−.055	.956			
	Smoking history	3.189	.177	2.200	**.029**			
	HAMD_24_	.124	.168	1.689	.093			
	HAMA_14_	.001	.001	.007	.995			
Dependence	Age	−.025	−.051	−.615	.539	.040	.985	.444
	Gender	1.814	.188	2.234	**.027**			
	Education	−.007	−.005	−.064	.949			
	BMI	.032	.020	−.256	.798			
	Smoking history	.311	.019	.234	.815			
	HAMD_24_	.077	.118	1.147	.253			
	HAMA_14_	−.065	−.084	−.807	.421			
Self-determination attitude	Age	.045	.077	.956	.341	.112	2.970	**.006**
	Gender	2.937	.259	3.192	**.002**			
	Education	.229	.141	1.780	.077			
	BMI	−.076	−.041	−.538	.591			
	Smoking history	3.412	.181	2.267	**.025**			
	HAMD_24_	.089	.116	1.168	.245			
	HAMA_14_	−.191	−.209	−2.099	**.037**			

Bold values indicate statistical significance; BMI, body mass index; HAMD_24_, 24-item Hamilton Rating Scale for Depression; HAMA_14_, 14-item Hamilton Anxiety Rating Scale; MDD, major depressive disorder; C-DAS-A, Chinese version of the dysfunctional attitude scale—form A.

## Discussion

To the best of our knowledge, this is the first study to investigate gender differences in dysfunctional attitudes in Chinese adult MDD patients. First, we found that MDD patients showed significantly higher C-DAS-A scores than HCs. Second, we observed significant gender differences in C-DAS-A total and three-factor scores (seeking applause, dependence, and self-determination attitude) in MDD patients, with women scoring higher than men. Third, five variables (age, gender, smoking history, HAMD_24_, and HAMA_14_) had predictive effects on and gender showed greatest contributions to C-DAS-A total and three-factor scores (seeking applause, dependence, and self-determination attitude).

### Gender Differences in Dysfunctional Attitudes in MDD Patients

Consistent with our hypothesis, our results revealed that females with MDD showed more dysfunctional attitudes in seeking applause, dependence, and self-determination attitude factors than male patients. Our results are also partially in line with the findings of two previous studies (19, 20). One study demonstrated that depressed women are associated with more dysfunctional attitudes in the dependence factor (19). The other study found that women with hypertension comorbid with depression show more dysfunctional attitudes in five factors of DAS (attraction and repulsion, seeking applause, compulsion, dependence, and self-determination attitude) than men (20). Although specific factors showing differences vary across different studies, these studies together with our study all support gender differences in dysfunctional attitudes in depression.

The reason why females with MDD exhibit more dysfunctional attitudes than males is unclear, particularly in the context that no gender difference in the dysfunctional attitudes in HCs is observed, which is consistent with the previous finding that there is no gender difference in DAS score of college students (16). One possible explanation is that the gender differences in dysfunctional attitudes in MDD may be associated with gender differences in neuroticism, which is a personality more commonly seen in females than in males (27–31). Previous studies have demonstrated that dysfunctional attitudes are closely linked to neuroticism (8, 32–35). Some items of DAS, such as “If you don’t have other people to lean on, you are bound to be sad” and “If I fail at my work, then I am a failure as a person”, reflect thinking styles commonly seen in subjects with neuroticism personality. Moreover, neuroticism has a close relationship with depression (36–38). We hypothesized that in depression, the depressed mood may interact with neuroticism and bring about more dysfunctional attitudes in females (39), particularly in the attitudes closely related to neuroticism, like seeking applause, dependence, and self-determination attitude. Although the specific factors showing differences vary across subgroups of patients with different severity, these results all support our main argument that there are gender differences in dysfunctional attitudes in patients with MDD.

An understanding of gender differences in dysfunctional attitudes in MDD may facilitate our understanding of and guide our treatment for depression in clinical practice. On one hand, the gender differences in dysfunctional attitudes in MDD may contribute to the gender differences in the development and prognosis of MDD. On the other hand, the gender differences in dysfunctional attitudes in MDD call for gender-specific interventional approaches in the treatment of depression, particularly in the interventions targeting the distorted cognition, like cognitive behavioral therapy (CBT). Our results suggest that CBT therapists should pay greater attention to the dysfunctional attitudes when working with female MDD patients.

### The Predictive Variables for Dysfunctional Attitudes in MDD Patients

Although each regression model accounted for only about 10% of the variance for dysfunctional attitudes, it had pointed out that age, gender, smoking history, HAMD_24_, and HAMA_14_ had an association with dysfunctional attitudes. Particularly, gender made the greatest contribution in each regression model, which is consistent with our finding that female MDD patients have higher DAS scores. As shown in the model, age had a negative prediction for the score of seeking applause, which is inconsistent with the demographic information of this study that female patients were older than males while with higher seeking applause score. The reason for consistency is unknown, possibly because the role of gender in seeking applause is greater than that of age.

Meanwhile, smoking history is an important predictor for dysfunctional attitudes, which is consistent with a previous study reporting worse cognitive function in smokers (40). However, the predictive effect of smoking history is relatively small and the results of subgroup analyses revealed no significant difference in C-DAS-A total score between MDD patients with and without a smoking history. The HAMD_24_ total score had a positive prediction for dysfunctional attitudes, which is consistent with previous studies (20, 24, 41) showing a close relationship between dysfunctional attitudes and severity of depression. We also found that the HAMA_14_ total score had a negative correlation with the self-determination attitude. Considering the relatively small predictive effects of these variables, future studies are needed to investigate additional effective predictive variables for dysfunctional attitudes.

### Limitations

There are a few limitations in this study and caution should be raised when interpreting the results of this study. First, the investigative data represent only the adult population of China in the age range of 18–55. It remains unknown whether the findings are suitable for geriatric or adolescent MDD patients. Second, the number of subjects is relatively small and the area distribution of participants is concentrated, which questions the representativeness of this sample for the general Chinese population. Thus, future studies should recruit geriatric or adolescent participants and more subjects from broader areas. Third, we are unable to provide the exact reason why DAS-A score of females with MDD is higher than that in males. Future studies investigating the mechanism of gender differences in dysfunctional attitudes in MDD patients are needed. Fourth, the recruitment of this study spanned 6 years, which means that the subjects of the same age may represent different generations and cognitive backgrounds. Shorten the duration of enrollment will deserve consideration in the future.

### Conclusions

There are gender differences in dysfunctional attitudes in MDD. Female patients have more dysfunctional attitudes than male patients, particularly in seeking applause, dependence, and self-determination attitude. A better understanding of the gender differences in dysfunctional attitudes in MDD may be useful for understanding the gender differences in clinical features of MDD and for developing gender-specific interventional approaches in the future.

## Data Availability Statement

The datasets generated for this study are available on request to the corresponding authors.

## Ethics Statement

The studies involving human participants were reviewed and approved by the Ethics Committee of the Second Xiangya Hospital of Central South University and the Ethics Committee of the Zhumadian Psychiatric Hospital. The patients/participants provided their written informed consent to participate in this study.

## Author Contributions

LL and BL co-designed the topic. JS, MW, XL, QD, LZ, JL, YJ, PW, HG, FZ, and YZ are responsible for participant recruitment and data collection. XQ and JS undertook the statistical analyses and wrote the initial draft of the manuscript. BL contributed substantial revisions to the manuscript.

## Funding

This study was supported by the National Science and Technologic Program of China (2015BAI13B02), the Defense Innovative Special Region Program (17-163-17-XZ-004-005-01), the National Natural Science Foundation of China (81171286, 91232714 and 81601180).

The funding sources had no role in the study design, data collection and analysis, interpretation of the data, preparation and approval of the manuscript, and decision to submit the manuscript for publication.

## Conflict of Interest

The authors declare that the research was conducted in the absence of any commercial or financial relationships that could be construed as a potential conflict of interest.
