# Corrigendum: Gender Differences in Dysfunctional Attitudes in Major Depressive Disorder

**DOI:** 10.3389/fpsyt.2020.541955

**Published:** 2020-09-16

**Authors:** Xuemei Qin, Jinrong Sun, Mi Wang, Xiaowen Lu, Qiangli Dong, Liang Zhang, Jin Liu, Yumeng Ju, Ping Wan, Hua Guo, Futao Zhao, Yan Zhang, Bangshan Liu, Lingjiang Li

**Affiliations:** ^1^ Department of Psychiatry, The Second Xiangya Hospital, Central South University, Changsha, China; ^2^ Mental Health Institute of Central South University, China National Clinical Research Center on Mental Disorders (Xiangya), China National Technology Institute on Mental Disorders, Hunan Technology Institute of Psychiatry, Hunan Key Laboratory of Psychiatry and Mental Health, Changsha, China; ^3^ Affiliated WuTaiShan Hospital of Medical College of Yangzhou University, Yangzhou Mental Health Centre, Yangzhou, China; ^4^ Department of Psychiatry, Zhumadian Psychiatric Hospital, Zhumadian, China

**Keywords:** major depressive disorder, dysfunctional attitudes, gender differences, seeking applause, dependence, self-determination attitude, gender-specific interventions

In the original article, there was a mistake in **Table 2** as published. A *P*-value was missing in **Table 2**, on the row “Compulsion”. The corrected **Table 2** appears below. Meanwhile, the full name showed as “14-item Hamilton rating scale for depression” of the HAMA_14_ was wrong in the title of Table 4. The corrected title of Table 4 is as following: Multiple linear regression analyses for assessing effects of age, gender, education, BMI, smoking history, 24-item Hamilton rating scale for depression (HAMD_24_), and 14-item Hamilton anxiety rating scale (HAMA_14_) predicting dysfunctional attitudes in major depressive disorder (MDD) patients.

We apologize for these errors and these do not change the scientific conclusions of the article in any way. The original article has been updated.

**Table 2:** Analysis of covariance (ANCOVA) of Chinese version of the dysfunctional attitude scale – form A (C-DAS-A) total and factor scores of major depressive disorder (MDD) and healthy control (HC) groups.

Item	MDD		HC		*P*
	Male (n = 75)	Female (n = 97)	Male (n = 74)	Female (n = 85)	
**Total score**	150.17 ± 25.98	160.05 ± 28.98	126.92 ± 29.94	123.73 ± 22.30	**<.001**
**Factor scores**					
Vulnerability	17.25 ± 4.42	18.72 ± 4.67	14.68 ± 4.95	14.81 ± 4.13	**<.001**
Attraction and repulsion	18.12 ± 5.04	18.53 ± 6.25	12.77 ± 5.26	12.93 ± 4.76	**<.001**
Perfectionism	18.21 ± 5.29	19.19 ± 5.94	15.15 ± 5.96	14.64 ± 4.06	**<.001**
Compulsion	18.28 ± 4.50	19.55 ± 4.62	16.15 ± 4.30	16.34 ± 3.71	**<.001**
Seeking applause	18.45 ± 5.16	20.40 ± 5.47	16.74 ± 8.41	15.59 ± 4.91	**<.001**
Dependence	18.67 ± 4.00	20.36 ± 5.23	15.78 ± 5.09	14.86 ± 4.15	**<.001**
Self-determination attitude	21.07 ± 5.95	23.03 ± 5.28	18.49 ± 5.53	17.24 ± 4.62	**<.001**
Cognition philosophy	20.12 ± 5.08	20.28 ± 5.62	17.16 ± 5.37	17.33 ± 4.73	**<.001**

Data are presented as mean ± SD; Bold values indicate statistical significance; P showed the difference in the C-DAS-A total and factor scores between MDD and HC groups; ANCOVA, analysis of covariance; C-DAS-A, Chinese version of the dysfunctional attitude scale – form A; MDD, major depressive disorder; HC, healthy control.

