# Supplementary material for: Gender Differences in Dysfunctional Attitudes in Major Depressive Disorder
Source: Front Psychiatry. 2020 Feb 27;11:86. doi: 10.3389/fpsyt.2020.00086 (PMC7057763; doi:10.3389/fpsyt.2020.00086)
Supplement: Supplementary file 1 [file DataSheet_1.docx]

Table S1. Demographic and clinical information of subgroups of MDD patients.

| Item | Severity of MDD | | | | |  | Comorbid with GAD | | |  | Smoking history | | |
| --- | --- | --- | --- | --- | --- | --- | --- | --- | --- | --- | --- | --- | --- |
|  | Moderate  (n = 114) | Severe  (n = 58) | *P* |  | MDDA  (n = 126) | | | MDDO  (n = 46) | *P* |  | With  (n = 17) | without  (n = 155) | *P* |
| Age (years) | 33.90 ± 10.36 | 37.48 ± 7.70 | **.012** |  | 35.16 ± 8.82 | | | 34.98 ± 11.80 | .925 |  | 35.13 ± 9.92 | 34.94 ± 7.28 | .940 |
| Education (years) | 10.49 ± 3.44 | 9.81 ± 3.54 | .226 |  | 10.06 ± 3.48 | | | 10.83 ± 3.45 | .200 |  | 10.15 ± 3.45 | 11.24 ± 3.72 | .226 |
| BMI (kg / m^2^) | 21.87 ± 3.01 | 22.28 ± 3.11 | .404 |  | 21.76 ± 3.06 | | | 22.67 ± 2.94 | .083 |  | 21.86 ± 3.00 | 23.39 ± 3.18 | **.048** |
| First episode age (years) | 30.20 ± 10.68 | 34.94 ± 8.25 | **.002** |  | 31.84 ± 9.39 | | | 31.70 ± 12.12 | .944 |  | 31.86 ± 10.36 | 31.25 ± 8.24 | .814 |
| Smoking history (person) | 13 | 4 | .349 |  | 13 | | | 4 | .752 |  | - | - | - |
| HAMD_24_ | 27.49 ± 4.45 | 39.76 ± 4.43 | **<.001** |  | 33.44 ± 7.02 | | | 26.65 ± 5.62 | **<.001** |  | 31.72 ± 7.50 | 30.82 ± 5.47 | .692 |
| HAMA_14_ | 15.81 ± 4.68 | 23.18 ± 5.91 | **<.001** |  | 20.96 ± 4.87 | | | 11.00 ± 2.32 | **<.001** |  | 18.38 ± 6.32 | 17.59 ± 4.98 | .659 |
| C-DAS-A total score | 154.92 ± 28.15 | 157.36 ± 28.09 | .515 |  | 156.06 ± 29.04 | | | 154.87 ± 25.49 | .806 |  | 156.68 ± 28.03 | 147.18 ± 27.82 | .185 |

Note: Abbreviations: MDD: major depressive disorder; GAD: Generalized anxiety disorder; MDDA: MDD with Anxiety group; MDDO: MDD only group; BMI: Body mass index; HAMD_24_: 24-item Hamilton Rating Scale for Depression; HAMA_14_: 14-item Hamilton Anxiety Rating Scale; C-DAS-A: Chinese version of the dysfunctional attitude scale – form A. Two-sample independent t-tests and chi-square tests were used to assess the differences in the demographic information between subgroups of MDD patients; Analyses of covariance (ANCOVA) were performed to assess the differences in the clinical information in different subgroups with unbalanced demographics as covariance; data are presented as Mean ± SD; There was no significant statistical difference in C-DAS-A total score between subgroups.

Table S2. Demographic and clinical information of different severity of MDD patients and HC groups.

| Item | Moderate MDD  (n = 114) | Severe MDD  (n = 58) | HC  (n = 159) | *P_1_* | *P_2_* |
| --- | --- | --- | --- | --- | --- |
| Age (years) | 33.90 ± 10.36 | 37.48 ± 7.70 | 34.55 ± 9.12 | .584 | **.030** |
| Sex (male) | 54 | 21 | 74 | .893 | .174 |
| Education (years) | 10.49 ± 3.44 | 9.81 ± 3.54 | 11.10 ± 3.59 | .161 | **.020** |
| BMI (kg / m^2^) | 21.87 ± 3.01 | 22.28 ± 3.11 | 23.64 ± 2.98 | **<.001** | **.004** |
| HAMD_24_ | 27.49 ± 4.45 | 39.76 ± 4.43 | 1.60 ± 2.35 | **<.001** | **<.001** |
| HAMA_14_ | 15.81 ± 4.68 | 23.18 ± 5.91 | 1.38 ± 2.09 | **<.001** | **<.001** |
| C-DAS-A total score | 154.92 ± 28.15 | 157.36 ± 28.09 | 125.21 ± 26.10 | **<.001** | **<.001** |

Note: Abbreviations: MDD: major depressive disorder; HC: healthy control; BMI: Body mass index; HAMD_24_: 24-item Hamilton Rating Scale for Depression; HAMA_14_: 14-item Hamilton Anxiety Rating Scale; C-DAS-A: Chinese version of the dysfunctional attitude scale – form A. Two-sample independent t-tests and chi-square tests were used to assess the differences in the demographic information between different severity of MDD patients and healthy controls; Analyses of covariance (ANCOVA) were performed to assess the differences in the clinical information between different severity of MDD patients and healthy controls with unbalanced demographics as covariance; data are presented as Mean ± SD; *P_1_*_:_ statistical significance of moderate MDD group and HC group; *P_2_*_:_ statistical significance of severe MDD group and HC group.

Table S3. Factorial ANOVA of gender and diagnosis on C-DAS-A total and factor scores in patients with moderate MDD and HC groups.

| Item | Main effects of diagnosis | | |  | | Main effects  of gender | | | |  | | Interaction  effects  (gender & diagnosis) | | | | |  | | | | Simple effects of gender | | | | | |
| --- | --- | --- | --- | --- | --- | --- | --- | --- | --- | --- | --- | --- | --- | --- | --- | --- | --- | --- | --- | --- | --- | --- | --- | --- | --- | --- |
|  | F | | *P* | |  | | F | | *P* |  | | | | F | *P* | | |  | | MDD  *P* | | | HC  *P* | | |  |
| **Total score**  **Factor scores** | 70.622 | **<.001** | | | |  | | .085 | .770 |  | | 2.644 | | | | .104 |  | | **-** | | | - | | |  |  |
| vulnerability | 37.653 | **<.001** | | | |  | | 1.948 | .164 |  | | 1.080 | | | | .300 |  | | - | | | - | | |  |  |
| attraction  and repulsion | 55.165 | **<.001** | | | |  | | .568 | .452 |  | | .002 | | | | .965 |  | | - | | | - | | |  |  |
| perfectionism  compulsion | 35.336  26.445 | **<.001**  **<.001** | | | |  | | .279  .113 | .598  .737 |  | | .982  .121 | | | | .323  .729 |  | | -  - | | | -  - | | |  |  |
| seeking applause | 10.661 | **.001** | | | |  | | .016 | .899 |  | | 1.491 | | | | .223 |  | | **-** | | | - | | |  |  |
| dependence | 50.010 | **<.001** | | | |  | | .024 | .877 |  | | 3.734 | | | | .054 |  | | **-** | | | - | | |  |  |
| self-determination attitude | 53.084 | **<.001** | | | |  | | .460 | .498 |  | | 10.023 | | | | **.002** |  | | **.011** | | | .070 | | |  |  |
| cognition philosophy | 10.553 | **<.001** | | | |  | | .030 | .863 | |  | | .003 | | | .956 |  | | | | - | | | - |  |  |

Note: ANOVA: analysis of variance; C-DAS-A: Chinese version of the dysfunctional attitude scale – form A; MDD: major depressive disorder; HC: healthy control. Factorial ANOVA of gender and diagnosis was performed with age, education, and BMI controlled.

Table S4. Factorial ANOVA of gender and diagnosis on C-DAS-A total and factor scores in patients with severe MDD and HC groups.

| Item | Main effects of diagnosis | | |  | | Main effects  of gender | | | |  | | Interaction  effects  (gender & diagnosis) | | | | |  | | | | Simple effects of gender | | | | | |
| --- | --- | --- | --- | --- | --- | --- | --- | --- | --- | --- | --- | --- | --- | --- | --- | --- | --- | --- | --- | --- | --- | --- | --- | --- | --- | --- |
|  | F | | *P* | |  | | | F | *P* |  | | | F | | | *P* | |  | | MDD  *P* | | | HC  *P* | | |  |
| **Total score**  **Factor scores** | 46.425 | **<.001** | | | |  | .570 | | .451 |  | | | 4.323 | | **.039** | |  | | .099 | | | .244 | | |  |  |
| vulnerability | 15.627 | **<.001** | | | |  | .937 | | .334 |  | | | .781 | | .378 | |  | | - | | | - | | |  |  |
| attraction  and repulsion | 48.566 | **<.001** | | | |  | .095 | | .759 |  | | | .786 | | .376 | |  | | - | | | - | | |  |  |
| perfectionism  compulsion | 19.430  8.842 | **<.001**  **.003** | | | |  | .041  2.004 | | .839  .158 |  | | | .341  6.505 | | .560  **.011** | |  | | **-**  **.021** | | | -  .336 | | |  |  |
| seeking applause | 15.971 | **<.001** | | | |  | 1.434 | | .232 |  | | | 4.422 | | **.037** | |  | | .054 | | | .444 | | |  |  |
| dependence | 28.479 | **<.001** | | | |  | .543 | | .462 |  | | | 4.509 | | **.035** | |  | | .096 | | | .222 | | |  |  |
| self-determination attitude | 15.871 | **<.001** | | | |  | .525 | | .470 |  | | | 2.658 | | .105 | |  | | - | | | - | | |  |  |
| cognition philosophy | 13.390 | **<.001** | | | |  | .006 | | .940 | |  | | | .036 | .850 | |  | | | | - | | | - |  |  |

Note: ANOVA: analysis of variance; C-DAS-A: Chinese version of the dysfunctional attitude scale – form A; MDD: major depressive disorder; HC: healthy control. Factorial ANOVA of gender and diagnosis was performed with age, education, and BMI controlled.
